# Effectiveness of Breast Milk Odor on Preterm Infant Feeding Cues: Protocol for a Randomized Clinical Trial

**DOI:** 10.2196/89618

**Published:** 2026-09-22

**Authors:** Dewi Irianti, Fajar Tri Waluyanti, Mega Hasanul Huda, Dessie Wanda

**Affiliations:** 1 Department of Pediatric Nursing, Universitas Indonesia, Pondok Cina, DepokWest Java, 16424, Indonesia

**Keywords:** breast milk odor, feeding cues, neonatal intensive care unit, olfactory stimulation, preterm infants, time to first successful feeding

## Abstract

**Background:**

Preterm infants often experience physiological instability and immature feeding coordination, which can delay the transition to oral feeding and prolong hospitalization. Nonpharmacological interventions are increasingly used to support feeding readiness and neurobehavioral regulation. Exposure to breast milk odor has shown potential benefit. However, robust evidence from randomized controlled trials using validated outcome measures remains limited, particularly in the Indonesian context.

**Objective:**

This study aims to evaluate the effectiveness of breast milk odor on feeding cues and time to first successful breastfeeding in preterm infants admitted to the neonatal intensive care unit (NICU).

**Methods:**

This study is a single-blind randomized controlled trial currently underway with 2 parallel groups involving 96 preterm infants (gestational age 32‐34 weeks) admitted to the NICU. Participants are randomly assigned to receive either breast milk odor exposure or a neutral odor control intervention. The intervention is administered 3 times daily for 3 consecutive days before feeding sessions. Feeding cues (the primary outcome) are assessed using the validated Feeding Cues Follow-Up Form (FCF-UF) during each feeding session (9 assessments over the 3-day intervention period). Time to first successful breastfeeding is assessed daily from day 4 until successful breastfeeding is achieved, or for a maximum follow-up of 10 days. Statistical analysis will be conducted after data collection is completed, with significance set at *P*<.05.

**Results:**

This study was funded in May 2025. Recruitment commenced in December 2025 and was completed in March 2026. A total of 104 participants were enrolled. Data collection was completed in April 2026, and data cleaning and statistical analysis were completed in August 2026. The study findings are expected to be published in 2026.

**Conclusions:**

This trial will provide evidence on whether breast milk odor is an effective, low-cost intervention to improve feeding readiness and facilitate earlier breastfeeding initiation in preterm infants.

## Introduction

Prematurity remains a leading cause of neonatal morbidity and mortality worldwide. Globally, approximately 10.7% of all live births are preterm, and more than 6.7% of these infants die from complications related to prematurity [1]. Indonesia is among the countries with the highest preterm birth rates, contributing significantly to the burden in Southeast Asia [2]. In addition to respiratory complications and infections, delayed initiation of oral feeding is a major clinical challenge in preterm infants, often resulting in prolonged neonatal intensive care unit (NICU) stays, impaired growth, and increased psychosocial and economic burden for families [3].

Preterm infants frequently experience difficulties in initiating oral feeding due to immature oromotor reflexes and uncoordinated sucking, swallowing, and breathing patterns, leading to ineffective feeding and reduced tolerance [4]. These feeding difficulties are associated with physiological instability, including oxygen desaturation and bradycardia, and often prolong dependence on orogastric tube feeding, thereby delaying the transition to independent oral feeding and limiting early mother and infant interaction [5,6].

Developmental care approaches in the NICU emphasize optimization of the sensory environment to support neurodevelopment and feeding readiness. Among these, olfactory stimulation has gained increasing attention, as the olfactory system is one of the earliest sensory modalities to mature. Fetuses can detect and learn odors from amniotic fluid as early as the second trimester, facilitating early sensory learning [7]. Breast milk contains bioactive volatile compounds, including aldehydes, ketones, and fatty acid derivatives, which produce a distinctive and biologically relevant odor that varies between mothers and across lactation stages [8].

Recent evidence suggests that exposure to breast milk odor may enhance feeding readiness and behavioral responses in preterm infants. Studies [9] have reported significant improvements in feeding cues, earlier transition from tube to oral feeding, and improved abdominal perfusion following breast milk odor exposure. In addition, breast milk odor has been associated with reduced stress responses, improved physiological stability, decreased pain responses during procedures, and calming behavioral effects [10-12]. Improvements in respiratory outcomes, such as reduced apnea frequency, and enhanced weight gain have also been reported [13,14].

Although several previous studies have reported beneficial effects of breast milk odor on feeding behavior and physiological stability, many were limited by small sample sizes, single-center designs, nonrandomized methods, or inconsistent intervention procedures. In addition, the timing, frequency, and duration of olfactory stimulation varied substantially across studies, making direct comparison difficult.

Despite encouraging preliminary findings, the current body of evidence remains limited by substantial heterogeneity in study designs, including variations in intervention protocols (eg, timing, duration, and delivery methods of odor exposure); outcome measures; and sample characteristics. Although recent meta-analyses indicate a positive effect of olfactory and gustatory stimulation on feeding readiness, many included studies are constrained by small sample sizes, lack of adequate blinding, and potential bias, limiting the strength and generalizability of the conclusions [4]. Accordingly, additional randomized controlled trials using standardized methods are warranted.

While fully blinded randomized controlled trials are considered the gold standard to minimize bias, their implementation in NICU settings can be challenging due to the nature of sensory-based interventions. Therefore, this study adopts a single-blind randomized controlled design, ensuring methodological rigor while maintaining feasibility in clinical practice. The conceptual framework of this study is based on the premise that olfactory stimulation through breast milk odor can activate early sensory pathways, enhance feeding-related behavioral cues, and improve physiological stability, thereby facilitating the transition to oral feeding in preterm infants [7,9].

To our knowledge, no previous multicenter randomized controlled trial has evaluated breast milk odor using a standardized intervention protocol together with repeated feeding cue assessments using a validated instrument, Feeding Cues Follow-Up Form (FCF-UF), in the Indonesian NICU setting. This study is expected to provide context-specific evidence that may inform neonatal nursing practice in similar resource-limited settings.

This study aims to evaluate the effects of breast milk odor stimulation on feeding cues and time to first successful breastfeeding in preterm infants admitted to the NICU. We hypothesize that preterm infants receiving breast milk odor exposure will demonstrate significantly higher feeding cue scores and achieve successful breastfeeding earlier than infants receiving the neutral odor control intervention. The findings may provide evidence to guide neonatal nursing practice, particularly in resource-limited settings.

## Methods

### Design

**Figure 1.**
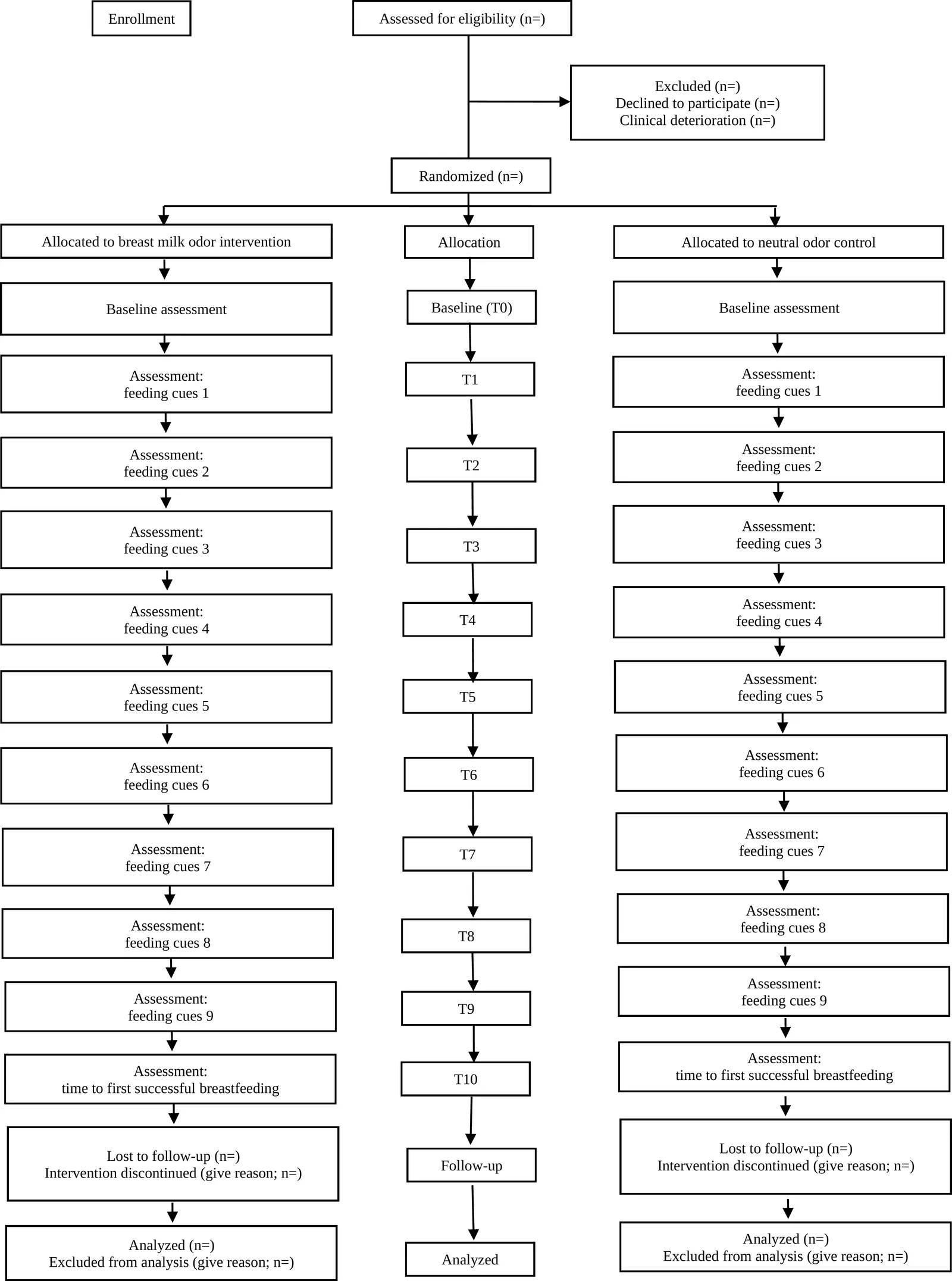
Study flow diagram of the randomized controlled trial protocol. T0 indicates the baseline assessment; T1-T9 represent the 9 feeding cue assessment sessions conducted during the 3-day intervention period; T10 indicates the assessment of time to first successful breastfeeding.

This study is a multicenter, single-blind randomized controlled trial with 2 parallel groups. This study protocol was developed in accordance with the SPIRIT (Standard Protocol Items: Recommendations for Interventional Trials) 2025 Statement (Checklist 1), and the trial will be conducted and reported following the CONSORT (Consolidated Standards of Reporting Trials) 2025 Statement. This design allows prospective assessment of methodological rigor, scientific integrity, and ethical standards, as well as a retrospective validation of study implementation and reporting [15,16]. The overall study procedures are summarized in Figure 1.

### Setting

This study was conducted in the NICUs of 2 tertiary referral hospitals in Central Java, Indonesia: Dr. Sardjito General Hospital in Yogyakarta and Dr. Soeradji Tirtonegoro General Hospital in Klaten. These NICUs were selected because they provide comparable standards of care for preterm infants, are equipped with comprehensive intensive monitoring facilities, and maintain a high rate of breastfeeding among preterm infants admitted to their units. Moreover, the selection was based on the high rate of preterm births in Central Java, ranging from 6% to 7% [17]. As tertiary referral hospitals, both NICUs manage a large volume of preterm infant admissions. Participants were recruited consecutively in both NICUs.

In both participating NICUs, preterm infants are initially fed via an orogastric or nasogastric tube according to standard neonatal care protocols. The transition to oral feeding is initiated when infants demonstrate physiological stability, coordinated suck-swallow-breath patterns, and feeding readiness as determined by the attending neonatologist and neonatal nurses. Once these criteria are met, breastfeeding attempts are routinely performed at each scheduled feeding session. During the study period, routine feeding management, including decisions regarding feeding advancement and breastfeeding initiation, will continue according to the standard clinical protocol in both the intervention and control groups. Breast milk odor exposure is provided as an adjunct to, rather than a replacement for, routine feeding care.

Eligible infants were identified daily through routine clinical screening and medical record review by trained NICU nurses and research assistants to standardize participant screening and recruitment.

### Participants

Preterm infants with a gestational age between 32 and 34 weeks were selected as participants in this study. As sucking, swallowing, and breathing reflexes begin to develop during this period but are not yet fully mature, targeted intervention is required to accelerate readiness for oral feeding while minimizing clinical variability and the risk of severe complications, such as severe hypoxia, intraventricular hemorrhage, and necrotizing enterocolitis (NEC) [18].

Other inclusion criteria were a minimum birth weight of 1,000 g; a stable hemodynamic condition, including infants receiving continuous positive airway pressure (CPAP); and infants receiving CPAP were eligible if respiratory support remained stable and olfactory stimulation could be administered safely without interrupting CPAP therapy; and infants who had not yet initiated oral feeding to ensure a uniform baseline of oral feeding development, which allowed the effects of the breast milk odor intervention to be measured without confounding from prior feeding experience [19]. In addition, written informed consent was obtained from the mothers prior to selection.

The exclusion criteria included infants with congenital abnormalities that could impair feeding ability, such as esophageal atresia or cleft lip and/or palate, metabolic disorders, a history of seizures, severe hypoxia, grade III or IV intraventricular hemorrhage, NEC, or prior exposure to other odors that could potentially interfere with the consistency of the breast milk odor intervention during the study period.

The screening process was conducted by a NICU nurse or research assistant through a review of the infant’s medical records and observation of the infant’s clinical condition. Once an infant met all established criteria, the parents were provided with a complete explanation of the study’s purpose and procedures, and informed consent was obtained before the infant was enrolled in the clinical trial. This criterion was intended to reduce baseline variability in feeding readiness. Recruitment followed a consecutive sampling approach, in which all eligible infants during the study period were approached to minimize selection bias.

### Sample Size Calculation

The sample size was calculated using G*Power version 3.1 based on an independent 2-group comparison (2-tailed independent-sample *t* test). Because previous studies investigating breast milk odor in preterm infants reported heterogeneous interventions, outcome measures, and effect estimates that were not directly comparable with the present study, a medium standardized effect size (Cohen *d*=0.50) was selected in accordance with Cohen’s recommendations for clinical research [20]. Assuming a 2-sided significance level of .05 and a statistical power of 80%, the minimum required sample size was 86 infants. To account for an anticipated 10% attrition rate, the final target sample size was increased to 96 infants, with 48 participants allocated to each group.

### Randomization and Allocation

Randomization is performed using a 1:1 block randomization scheme with a fixed block size of 4 to ensure balanced group allocation. The allocation sequence was generated using a computer-generated randomization sequence by an independent statistician who was not involved in participant recruitment or outcome assessment.

Allocation concealment is maintained using sequentially numbered, sealed, opaque envelopes. Eligible participants are enrolled by a research assistant, who opens the next envelope in sequence only after enrollment to determine group assignment. This procedure ensures that allocation remains concealed until the point of assignment. Randomization was performed separately at each participating study site using the same allocation procedure to maintain balanced group assignment throughout the recruitment period.

### Blinding

This study used a single-blind design in which the outcome assessor is blinded to group allocation. Due to the nature of the intervention, complete blinding of caregivers and personnel administering the intervention was not feasible. However, efforts were made to minimize bias by standardizing procedures and ensuring that the assessor evaluating feeding cues was unaware of group assignments.

### Intervention

The intervention is initiated at the time of feeding preterm infants. For infants receiving CPAP, the olfactory stimulus is administered without interrupting respiratory support. A sterile gauze containing breast milk is positioned near the infant’s nose, outside the CPAP interface, allowing passive exposure to the odor without interfering with airflow or device function. This procedure was selected to maintain safety and permit intervention delivery during CPAP support. No modifications to the CPAP settings or interface were made during the intervention.

Prior to each intervention session, the infant’s respiratory condition is assessed to ensure adequate nasal patency. The intervention is performed only when there is no significant airway obstruction, excessive nasal secretions, or clinical instability that could interfere with olfactory perception. To minimize the influence of external odors, the intervention is administered immediately before scheduled feeding sessions in a consistent NICU environment. Wherever feasible, routine procedures involving strong odors, such as skin antisepsis or adhesive remover sprays, are completed before the intervention or delayed until after completion of odor exposure. Because complete elimination of environmental odors is not feasible in routine NICU practice, both the intervention and control groups receive identical routine clinical care under the same environmental conditions, thereby minimizing differential exposure to external odors.

One minute before feeding, breast milk odor was applied using sterile gauze placed near the infant’s nose and kept in place until feeding was completed. Feeding in this study is conducted according to standard NICU practice, primarily using intermittent bolus feeding via an orogastric tube. Breastfeeding attempts are introduced based on infant readiness as assessed by clinical staff. Each feeding session typically lasts approximately 15 to 30 minutes, depending on the infant’s tolerance and clinical condition.

The breast milk odor was administered 3 times daily for 3 consecutive days for a total of 9 feedings. An assessment of feeding cues was conducted using video recordings captured during the intervention. The videos were reviewed by a pediatric nursing specialist with at least 5 years of NICU clinical experience who was blinded to group allocation (intervention or control) using a feeding cue assessment instrument that had been tested for validity and reliability.

The breast milk used for olfactory stimulation was obtained from each infant’s own mother whenever available. Mothers were instructed to express breast milk using sterile techniques on the same day as the intervention. The expressed milk was labeled immediately after collection and stored at 2 °C to 4 °C for no longer than 24 hours before use. Prior to each intervention session, designated research personnel retrieved the breast milk from storage, allowed it to reach room temperature, and prepared the sterile gauze according to the standardized study protocol. In cases where maternal breast milk was unavailable or insufficient, donor breast milk was used with maternal consent.

The intervention is administered exclusively by trained research staff who are not involved in outcome assessment. The prepared breast milk was delivered to the intervention personnel immediately before each scheduled feeding session. All research staff received standardized training before study initiation to ensure consistent implementation of the intervention protocol across both study sites.

The 3-day intervention period was selected based on previous studies [9] and clinical evidence suggesting that short-term sensory stimulation in preterm infants can produce measurable effects on feeding behavior within 48 to 72 hours. Outcome measurements were conducted in parallel with the intervention sessions according to a predefined assessment schedule.

### Control Condition

The control group received an identical procedure using a clear, odorless, starch-based liquid designed to visually resemble breast milk while minimizing olfactory stimulation. The control fluid was prepared under sterile conditions and did not contain any volatile compounds associated with breast milk odor. The volume, timing, and duration of exposure were standardized and matched to those of the intervention group. The control condition was designed according to the principles of neutral olfactory control used in previous olfactory intervention studies to maintain procedural consistency while minimizing olfactory stimulation and sensory bias [21].

### Measures

The FCF-UF, originally developed by Yücel et al [9], is a structured instrument designed to assess feeding cues in preterm infants. The instrument evaluates behavioral state, rooting, sucking attempts, and physiological stability. The instrument consists of dichotomous items scored as either present (score 1) or absent (score 0). Individual item scores are summed to obtain a total feeding cue score, with higher total scores indicating greater feeding cues.

In this study, feeding cues were assessed using the Indonesian version of the FCF-UF, which was cross-culturally adapted and validated for use in the Indonesian NICU setting. During each feeding session, infants were video recorded, and the recordings were independently reviewed by a blinded pediatric nursing specialist with at least 5 years of NICU experience using the standardized FCF-UF scoring criteria. The Indonesian version demonstrated excellent internal consistency (Cronbach α=0.92), excellent test-retest reliability (intraclass correlation coefficient=0.99), and excellent construct validity based on confirmatory factor analysis (*χ*^2^_20_=17.4, root mean square error of approximation=0.000, comparative fit index=1.000, and Tucker-Lewis index=1.001) [22].

### Outcome Measures

The primary outcome of this study was feeding cues, as measured by the FCF-UF. Feeding cues were assessed 3 times daily during each feeding session over the 3-day intervention period (total of 9 assessments). The assessments were conducted using video recordings, which were evaluated by a blinded pediatric nursing specialist to ensure objectivity and reduce observer bias.

The secondary outcome was the time to first successful breastfeeding, defined as the duration (in minutes) from the onset of observable feeding cue behaviors until the infant achieved the first successful breastfeeding. Observable feeding cue behaviors included rooting, head turning toward the breast, mouth opening, and searching for the nipple following tactile stimulation. Successful breastfeeding is defined as effective latching onto the mother’s breast followed by coordinated sucking, swallowing, and breathing for a sustained period, as observed by trained clinical staff.

Although breastfeeding attempts may occur during routine clinical care during the 3-day intervention period, these routine attempts were not included in the assessment of the secondary outcome. To ensure a standardized observation period across all participants, the predefined secondary outcome (time to first successful breastfeeding) was prospectively measured beginning on day 4, after completion of the intervention. Observations were conducted daily for a maximum of 60 minutes per day and continued for up to 10 days or until successful breastfeeding was achieved.

This outcome was measured using a stopwatch starting from day 4 after completion of the intervention. The measurement of time to first successful breastfeeding therefore began after completion of the 3-day intervention period (day 4). Postnatal age and other relevant clinical variables were recorded and will be considered as potential confounders in the analysis.

All outcome data were recorded in structured electronic forms immediately after each assessment to ensure accuracy and completeness. The schedule of enrollment, intervention, and outcome assessments is presented in Table 1.

**Table 1.** Schedule of enrollment, intervention, and outcome assessments.

Study phases	Day 0	Days 1‐3	Days 4‐10
Screening and eligibility	✓		
Informed consent	✓		
Randomization	✓		
Intervention (breast milk odor exposure)		✓^a^	
Feeding cue assessment (FCF-UF^b^)		✓^c^	
Time to first successful breastfeeding			✓^d^

a Intervention administered 3 times daily during days 1-3.

b FCF-UF: Feeding Cues Follow-Up Form.

c Feeding cues assessed 3 times daily during days 1-3 (9 assessments in total).

d Assessed daily from day 4 until successful breastfeeding or for a maximum of 10 days.

### Data Management Plan

All data were recorded in electronic forms and stored on the principal investigator’s password-protected computer, accessible only to the principal investigator. Identity data were stored separately from the research data. Only the principal investigator had full access to the complete dataset.

### Safety Considerations

Breast milk odor stimulation is a safe and noninvasive intervention. Infants were monitored continuously during the intervention. If any deterioration occurred (eg, desaturation, bradycardia, or apnea), the intervention was discontinued and the infant was cared for according to NICU standards.

### Ethical Considerations

This study was approved by the Research Ethics Committee of the Faculty of Nursing, Universitas Indonesia (KET-007/UN2.F12.D1.2.1/PPM.00.02/2025), and by the Medical and Health Research Ethics Committee, Faculty of Medicine, Public Health, and Nursing, Universitas Gadjah Mada Dr. Sardjito General Hospital (KE/FK/0979/EC/2025). The study was prospectively registered with the Indonesia Clinical Research Registry (INA-CRR) under registration number INA-5RCW7NLO on January 22, 2025, prior to participant recruitment [23].

Written informed consent was obtained from the parents of all participating infants prior to enrollment in the study. To ensure privacy and confidentiality, all collected data were deidentified and stored in secure, password-protected electronic systems accessible only to the principal investigator. Personal identifiers were removed from the dataset prior to analysis, and all procedures were conducted in accordance with applicable data protection standards. No financial or material compensation was provided to participants for participation in this study.

### Data Analysis

Data analysis will be conducted according to the intention-to-treat principle; therefore, all randomized participants will be included in the analysis according to their assigned groups, regardless of their adherence to the intervention. Outcomes will be analyzed using an independent *t* test for normally distributed data or the Mann-Whitney *U* test when normality assumptions are not met. If repeated measurements are available across multiple points, longitudinal analysis will be performed to more comprehensively assess response changes over time. For the secondary outcome of time to first successful breastfeeding, Kaplan-Meier survival analysis will be used, with between-group comparisons performed using the log-rank test. Infants who do not achieve successful breastfeeding within the 10-day observation period will be treated as censored observations. Intervention effects will be reported as the mean difference between groups, accompanied by effect sizes, using Cohen *d* to describe the magnitude of the intervention’s impact.

### Data Monitoring

Due to the minimal risks involved, a data monitoring committee was not established. The research team monitored all events throughout the study.

### Strategies for Improving Adherence

To ensure protocol adherence, the researchers implemented several monitoring strategies during the intervention. The intervention schedule—administered 3 times daily—was consistently maintained through coordination with the NICU nursing team, who provided reminders and helped ensure that each intervention was delivered according to the established timeline. In addition, feeding cues were assessed using the FCF-UF by trained observers, specifically pediatric nursing specialists with at least 5 years of NICU clinical experience, ensuring the quality and consistency of assessment throughout the study.

### Dissemination Plan

Study findings will be submitted to peer-reviewed journals and presented at scientific meetings. Results will also be shared with participating hospitals to inform local neonatal nursing practice.

## Results

This study was funded in May 2025. Recruitment commenced in December 2025 and was completed in March 2026, with a total of 104 participants enrolled. Baseline demographic and clinical characteristics, including gestational age, birth weight, and clinical stability, were collected for all participants. Data collection was completed in April 2026, followed by data cleaning and statistical analysis, which were completed in August 2026. No major deviations from the study protocol have been identified. The study findings are expected to be disseminated in 2026.

## Discussion

### Principal Findings

This study is designed to evaluate whether breast milk odor improves feeding cues and shortens the time to first successful breastfeeding in preterm infants. If beneficial, the intervention may represent a simple sensory-based strategy that can be integrated into routine NICU care.

### Comparison With Prior Work

Previous studies have consistently suggested that breast milk odor may improve feeding readiness and facilitate the transition to oral feeding in preterm infants. Recent systematic reviews and meta-analyses also reported beneficial effects on feeding outcomes, physiological stability, and selected clinical outcomes among preterm infants. However, these reviews also highlighted substantial heterogeneity in intervention protocols, outcome measures, participant characteristics, and methodological quality. Many included studies were conducted in single centers with relatively small sample sizes, limiting the strength and generalizability of the available evidence [4,6].

Among individual randomized trials, Yücel et al [9] demonstrated improvements in feeding cues and earlier transition to oral feeding following breast milk odor exposure, while Mohamed Soliman et al [13] reported enhanced feeding progression and behavioral responses. Despite these promising findings, differences in intervention delivery, assessment methods, and study design make direct comparisons difficult. These limitations underscore the need for standardized randomized controlled trials using validated outcome measures.

The present study was designed to address these gaps by using a multicenter randomized controlled design with concealed allocation, blinded outcome assessment, and a standardized breast milk odor protocol administered 3 times daily for 3 consecutive days. Unlike previous studies, feeding cues are assessed repeatedly using the validated FCF-UF, allowing comprehensive evaluation of behavioral changes over time. Conducting this study in 2 tertiary NICUs in Indonesia also provides context-specific evidence from a Southeast Asian setting where standardized developmental care interventions remain limited. Collectively, these methodological improvements are expected to strengthen the current evidence and support the integration of breast milk odor as a simple, low-cost intervention to promote feeding readiness in preterm infants.

### Strengths and Limitations

Strengths of this study include the randomized controlled design, standardized intervention procedures, and use of validated outcome measures. Blinded outcome assessment may also reduce measurement bias. Several limitations should also be considered. First, complete blinding is not feasible because of the nature of the intervention. Second, natural variation in breast milk composition between mothers may influence odor characteristics. Third, the study is conducted in selected NICUs, which may limit generalizability to other settings.

### Future Directions

Future research should examine longer-term outcomes, including feeding independence, growth, breastfeeding continuation, and neurodevelopment. Multicenter trials with larger and more diverse populations would further strengthen external validity.

## Supplementary material

10.2196/89618 Checklist 1 SPIRIT checklist.

## Abbreviations

CONSORT: Consolidated Standards of Reporting Trials
CPAP: continuous positive airway pressure
FCF-UF: Feeding Cues Follow-Up Form
NEC: necrotizing enterocolitis
NICU: neonatal intensive care unit
SPIRIT: Standard Protocol Items: Recommendations for Interventional Trials
